# Prophylactic platelet transfusion prior to central venous catheter placement in patients with thrombocytopenia: study protocol for a randomised controlled trial

**DOI:** 10.1186/s13063-018-2480-3

**Published:** 2018-02-20

**Authors:** Emma K. van de Weerdt, Bart J. Biemond, Sacha S. Zeerleder, Krijn P. van Lienden, Jan M. Binnekade, Alexander P. J. Vlaar, Study collaborative, A. van Leent, M. Koeman, P. F. Ypma, M. S. Arbous, A. M. P. Demandt, W. N. K. A. van Mook, J. H. M. Tordoir, E. Wolthuis, J. G. A. M. Blomjous, R. M. Determann, H. Endeman, E. D. Kerver, W. J. F. M. van der Velden, R. Vink, R. P. H. Bokkers, A. B. U. Mäkelburg, W. M. van den Bergh, P. R. Tuinman

**Affiliations:** 1 Department of Intensive Care Medicine, Academic Medical Centre, Meibergdreef 9, 1105 AZ Amsterdam, The Netherlands; 2 Laboratory of Experimental Intensive Care and Anaesthesiology (L.E.I.C.A.), Academic Medical Centre, Meibergdreef 9, 1105 AZ Amsterdam, The Netherlands; 3 Department of Radiology, Academic Medical Centre, Meibergdreef 9, 1105 AZ Amsterdam, The Netherlands; 4 Department of Haematology, Academic Medical Centre, Meibergdreef 9, 1105 AZ Amsterdam, The Netherlands; 5 G3–228; Department of Intensive Care, Academic Medical Centre, Meibergdreef 9, 1105 AZ Amsterdam, The Netherlands

**Keywords:** Central venous catheter, Thrombocytopenia, Platelet count, Bleeding complication

## Abstract

**Background:**

Severe thrombocytopenia should be corrected by prophylactic platelet transfusion prior to central venous catheter (CVC) insertion, according to national and international guidelines. Even though correction is thought to prevent bleeding complications, evidence supporting the routine administration of prophylactic platelets is absent. Furthermore, platelet transfusion bears inherent risk. Since the introduction of ultrasound-guided CVC placement, bleeding complication rates have decreased. The objective of the current trial is, therefore, to demonstrate that omitting prophylactic platelet transfusion prior to CVC placement in severely thrombocytopenic patients is non-inferior compared to prophylactic platelet transfusion.

**Methods/design:**

The PACER trial is an investigator-initiated, national, multicentre, single-blinded, randomised controlled, non-inferior, two-arm trial in haematologic and/or intensive care patients with a platelet count of between 10 and 50 × 10^9^/L and an indication for CVC placement. Consecutive patients are randomly assigned to either receive 1 unit of platelet concentrate, or receive no prophylactic platelet transfusion prior to CVC insertion. The primary endpoint is WHO grades 2–4 bleeding. Secondary endpoints are any bleeding complication, costs, length of intensive care and hospital stay and transfusion requirements.

**Discussion:**

This is the first prospective, randomised controlled trial powered to test the hypothesis of whether omitting forgoing platelet transfusion prior to central venous cannulation leads to an equal occurrence of clinical relevant bleeding complications in critically ill and haematologic patients with thrombocytopenia.

**Trial registration:**

Nederlands Trial Registry, ID: NTR5653 (http://www.trialregister.nl/trialreg/index.asp). Registered on 27 January 2016. Currently recruiting. Randomisation commenced on 23 February 2016.

**Electronic supplementary material:**

The online version of this article (10.1186/s13063-018-2480-3) contains supplementary material, which is available to authorized users.

## Background

Central venous catheter (CVC) placement is a frequently applied medical intervention that enables both monitoring and treatment of patients [1, 2]. The inserted cannula provides central venous access either in the neck region (subclavian vein or jugular vein) or groin region (femoral vein). CVCs form an essential element of treatment in various patient categories, mainly haematologic and intensive care patients [1, 2]. The latter patient categories have an elevated risk for a low platelet count (thrombocytopenia), due to their treatment and the physiopathology of their illnesses [3–5]. Thrombocytopenia is associated with an increased bleeding risk for many invasive procedures [6]. However, abnormal traditional coagulation tests are known to be a poor predictor of peri-procedural bleeding [7–9]. Current national and international guidelines are conflicting, most recent Dutch and UK guidelines support prophylactic platelet transfusion below a platelet count of 50 × 10^9^/L, prior to CVC placement [10, 11]. Others support the administration of prophylactic platelet transfusion below a platelet count of 20 × 10^9^/L; however, the evidence supporting these recommendations is of low quality [12, 13]. Current prophylactic platelet transfusion practice prior to CVC placement varies widely [14].

In the last decades, more has become known about transfusion-related morbidity and mortality; platelet transfusion bears a substantial risk for morbidity and mortality, including transfusion-related acute lung injury (TRALI), transfusion-associated cardiac overload (TACO), allergic reactions, allo-immunisation and transfusion-related infections [15–21]. In a prospective cohort study of consecutive intensive care unit (ICU) patients receiving blood transfusion products, 6% developed TACO [22]. In the Netherlands, 0.32% of pooled platelet concentrates and 0.23% of apheresis platelet concentrate units are contaminated with bacteria [23]. Transfusion of platelets is an independent risk factor for the onset of nosocomial infections in ICU patients, with a hazard ratio of 1.40 (95% confidence interval (CI) 1.2–1.8) [24]. Next to the burden of transfusion exposure, blood products are expensive and scarce. Critically ill and haematologic patients are at higher risk of transfusion-related morbidity and mortality [15, 25]. Altogether, these insights in transfusion-related morbidity and mortality have made platelet transfusion without a very strict indication less desirable.

Recent retrospective studies suggest that the experience of the physician and the technique used (ultrasound (US) vs. landmark) rather than the platelet count predicts bleeding complications [26–28]. The introduction of US guidance for CVC placement was a major improvement [29]. The use of US for CVC insertion results in a lower number of puncture attempts, arterial puncture, haematoma pneumothorax and haematothorax [29–31]. One meta-analysis showed a lower incidence of arterial puncture with the use of US, namely 37/2009 (1.8%), compared to 196/2018 (9.7%) with anatomical landmarks (RR 0.25, 95% CI 0.15 to 0.42). Also, the occurrence of haematoma was reduced from 113/1512 (7.5%) to 24/1500 (1.6%), with a RR 0.30 (95% CI 0.19 to 0.46) [31].

Consequently, recent retrospective studies suggest that US-guided central venous cannulation can safely be performed in patients with a platelet count above 20 × 10 ^9^/L [28, 32, 33]. In a study with 604 CVC placements, in 193 patients with acute leukemia, manual compression to stop bleeding was required in eight patients, no major bleeding was observed [28]. However, this study missed a control group and the administration of prophylactic platelet transfusion was not standardised, resulting in high risk of bias.

The current protocol describes the first randomised controlled trial to evaluate the effect of prophylactic platelet transfusion in haematologic and intensive care patients in need of a CVC.

## Methods/design

### Study hypothesis

We hypothesise that the improved standards of CVC placement, e.g. the use of US, make the need for correction of thrombocytopenia prior to CVC placement obsolete.

### Study objective

The primary objective is to demonstrate that the omission of prophylactic platelet transfusion in severely thrombocytopenic patients does not increase the amount of bleeding complications related to CVC placement.

### Primary endpoint

A procedure-related relevant bleeding, occurring within 24 h after the procedure. A WHO grade of 2–4 (Additional file 1) up to 24 h of randomisation is defined as relevant bleeding.

### Secondary endpoints

• Platelet transfusion requirements within 24 h of CVC placement

• Number of RBC transfusions within 24 h of CVC placement

• WHO grade-1 bleeding within 24 h of CVC placement

• Haematoma size

• Haemoglobin level at 1 h and 24 h after CVC placement

• Platelet transfusion increment

• HEME bleeding score (Additional file 1)

• Allergic transfusion reaction within 24 h

• Onset of acute lung injury within 48 h

• Length of hospital stay

• Costs

### Design

The PACER trial is a randomised controlled, non-inferiority trial on prophylactic platelet transfusion prior to CVC placement in patients with severe thrombocytopenia. It is an investigator-initiated, multicentre, parallel, randomised controlled, two-arm trial in haematologic and/or intensive care patients with thrombocytopenia and an indication for CVC placement. The PACER trial will be conducted according to the principles of the Declaration of Helsinki as stated in the current version of Fortaleza, Brazil, 2013 [34] and in accordance with the Medical Research Involving Human Subjects Act (WMO). The Institutional Review Board of the Academic Medical Centre, Amsterdam, the Netherlands, approved the trial protocol under reference number 2015_27#B201662. The trial is registered at http://www.trialregister.nl/trialreg/admin/rctview.asp?TC=5653 (NTR5653). Intensive care patients will be provisionally included under a strategy of deferred consent, which is explained in detail in the ‘Consent’ section below. For patients at the Department of Haematology, written informed consent is obtained prior to randomisation. We adhered to the SPIRIT statement (Additional file 2 SPIRIT checklist).

### CONSORT diagram

**Fig. 1:**
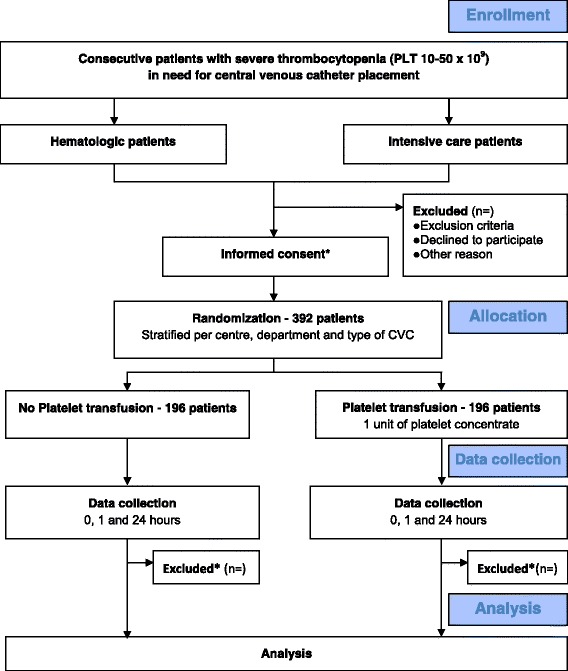
PACER trial (Consolidated Standards of Reporting Trials (CONSORT)) Diagram. CONSORT diagram of PACER. For haematologic patients, informed consent is required prior to randomisation. For the intensive care patients, randomisation takes place via deferred consent. If consent is not obtained, data are excluded

The Consolidated Standards of Reporting Trials (CONSORT) diagram of the PACER study is presented in Fig. 1. Consecutive thrombocytopenic patients who have an indication for central venous catheterisation are screened. Demographic data are registered regardless of meeting enrolment criteria using a predefined screen log. If patients are excluded for participation, the reason(s) for exclusion are registered. For the screening of thrombocytopenia, routinely conducted laboratory values are used. The indication for a CVC is determined at the physician’s discretion.

### Setting

The PACER trial is a multicentre study performed in six academic and five teaching hospitals in the Netherlands. We plan to include 392 patients in 36 months, with a potential limit of 462 patients to accommodate loss to follow-up and possible dropout.

### Study population

Thrombocytopenic patients with a platelet count of 10–50 × 10^9^ are eligible for participation if a central line is indicated. Both patients who need elective and emergency line insertions are appropriate for randomisation. Both tunnelled and non-tunnelled CVCs are suitable for inclusion, as well as lines inserted for continuous venovenous haemodiafiltration. Notably, patients requiring replacement of central catheters are also eligible. Patients can participate in the study multiple times; however, a patient can be randomised once per 24 h. The CVC should be expected to be in situ for at least 24 h to monitor 24-h bleeding complications. Therefore, central lines for single plasmapheresis or the harvesting of stem cells will not be included. All other indications can be included, e.g. inotrope medication, lack of peripheral venous access, haemodialysis, haemodynamic monitoring, administration of irritating medication such as chemotherapy. According to guidelines, patients with an International Normalised Ratio (INR) of > 1.5 before line placement are excluded [35]. However, patients are eligible after correction of an elevated INR with fresh frozen plasma or prothrombin concentrate. Non-adult patients (age below 18 years) are excluded, as are patients with a history of congenital or acquired coagulation factor deficiency or bleeding diathesis. Also patients on therapeutic anticoagulant therapy are excluded. Of note, patients with a single platelet aggregation inhibitor and/or therapeutic unfractionated heparin that is discontinued at least 1 h prior to insertion are considered to be eligible.

### Consent

Informed consent will be obtained from all participants or a legal representative in case the former is impossible. As mentioned previously, this study involves two patient categories. In haematologic patients, lines are usually inserted electively for the administration of chemotherapy. Therefore, informed consent will be obtained before line placement. For the intensive care patient category, obtaining informed consent prior to CVC insertion is often not feasible. Therefore, for the intensive care setting patients may be included using the deferred consent procedure. We will randomise each patient at the ICU who meets the inclusion criteria directly before CVC placement. Informed consent from the legal representative will be requested as soon as possible. If informed consent is denied by a legal representative, the patient is excluded and data will no longer be used. Thenceforth, the patient is transfused according to the policy of the attending physician. If a patient dies before informed consent can be obtained, the data are used [36].

### Randomisation and blinding

Randomisation will be performed using a dedicated, password-protected, SSL-encrypted website with ALEA® software (TenALEA consortium, Amsterdam, The Netherlands). The researcher randomises the patient. Random block sizes are used. Block randomisation will be stratified per centre and per patient population (ICU vs. haematologic patients). Tunnelled lines are stratified per centre. Also, lines placed for dialysis/CVVH are stratified from central lines placed for other indications. This stratification takes place since dialysis/CVVH catheters are larger-bore catheters, made of stiffer materials and bear increased risk for bleeding [37].

The proceduralist, the physician who inserts the catheter, is blinded for treatment allocation. This blinding takes place to prevent bias in ordering platelet transfusion in case of a peri-procedural bleeding complication. The proceduralist is not directly involved in patient care of the included patient. Blinding is not feasible for the patient and the treating medical staff. Obtained photographs of the insertion site will be analysed for haematoma size and bleeding post hoc. This provides an additional blinded bleeding outcome.

### Treatment arms

Included patients will be randomised into one of two groups. One group of patients will be allocated to not receiving platelet transfusion prior to placement of the CVC, this is the experimental group. The other group of patients will be transfused with 1 unit of platelet concentrate prior to placement of the catheter, this is the comparison group. All platelet products will be manufactured, screened and stored according to local Dutch standards, (Sanquin Blood Bank). Platelet concentrates are prepared from pooled buffy coats from five donors, and re-suspended in plasma, after which pooled platelet concentrates are leukoreduced by filtration. All platelet products are stored, with gentle agitation at 20–24 °C, for up to 7 days in the Netherlands. All other care will be according to standard practice as indicated by the treating physician.

### Central venous catheter placement

All CVCs will be inserted percutaneously, under real-time US guidance, using the Seldinger technique. In the transfusion arm, catheters will be inserted as soon as possible after administration of 1 unit of platelet concentrate. The type of US device, catheters and (local) anaesthesia used is according to local hospital protocols. The puncture location will be determined by the puncturing physician. All procedures will be performed by experienced physicians, e.g. at least 50 previous line placements [38]. Proceduralists can be experienced residents or consultant physicians in intensive care, anaesthesiology, haematology, (interventional) radiology and surgery. Procedural details, such as arterial puncture, vein lesion and number of puncture attempts are recorded.

### Bleeding complications

Bleeding complications will be scored using WHO grades of bleeding specified for CVC placement, which are defined in Additional file 1. The WHO bleeding score is adapted according to Zeidler [28]. Grade 1 consists of mild symptoms not requiring any intervention; for example, local haematoma formation or wound oozing. Grade 2 bleeding is defined as mild symptoms requiring interventions, without haemodynamic instability or red blood cell (RBC) transfusion. For the current study, this includes procedure-related bleeding that requires more than 20 min of manual compression to stop. Grade 3 bleeding is defined as procedure-related bleeding requiring red cell transfusion. Grade 4 bleeding is defined as bleeding associated with hemodynamic instability or death, defined as CVC-related bleeding associated with severe hemodynamic instability (hypotension; > 50 mmHg fall or > 50% decrease in either systolic or diastolic blood pressure, with associated tachycardia (heart rate increase of > 20% for 20 min) and requiring RBC transfusion over routine transfusion needs or fatal bleeding [39].

Furthermore, a distinction will be made between minor and major bleeding. Minor bleeding is defined as WHO bleeding scale grade 1 or 2. Major bleeding is defined as WHO grade 3 or 4 bleeding. The proceduralist can administer rescue platelets at clinical indication. Rescue platelet concentrate can be given irrespective of treatment allocation, for which the proceduralist remains blinded.

In case of post-procedural bleeding, physicians are encouraged to undertake the following steps:

1. Inspection of the insertion site

2. Apply manual compression, for a maximum duration of 20 min

3. Consider a skin suture at the insertion site

4. Consider rescue platelet transfusion

5. Consider the possibility of a radiological of surgical intervention

### Protocol violation

If platelet concentrate is administered to patients assigned to not receiving platelet concentrate, patients will not be excluded. Reasons for additional transfusion are noted. Patients will be analysed according to intention-to-treat and per-protocol analyses. If line placement is unsuccessful due to technical reasons, data will also be analysed according to the intention-to-treat analysis. If blinding of the proceduralist is violated, this will be noted; however, patient data will not be excluded. If the 24-h time point is not completed, due to a CVC that is in situ for less than 24 h, or in the case that a patient deceases within this timeframe, obtained data will be analysed using missing data for the 24-h time point.

### Participant timeline and study flowchart

**Fig. 2:**
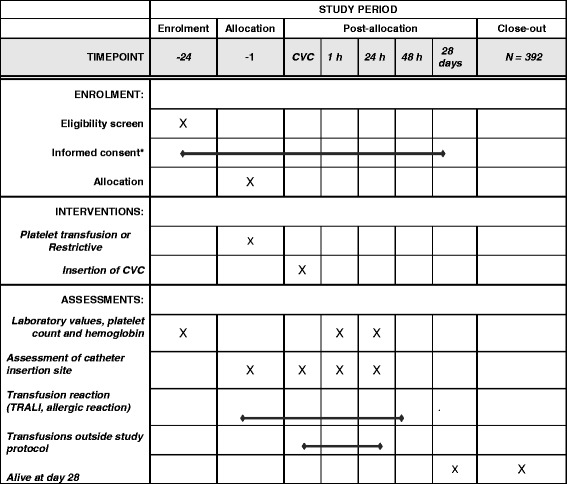
Informed consent will be obtained from all participants or a legal representative in case the former is impossible. Intensive Care patients may be included using the deferred consent procedure. We will randomise each patient at the intensive care unit (ICU) who meets the inclusion criteria directly before CVC placement. Informed consent from the legal representative will be requested as soon as possible. If a patient deceases before informed consent can be obtained, data is used

The participant timeline is presented in Fig. 2. The primary outcome of the study is procedure-related WHO bleeding grades 2–4, occurring within 24 h after the procedure.

Secondary endpoints are subdivided into clinical outcome variables and health-economic-outcome variables. WHO grade-1 bleedings and subdivision on minor and major bleeding are scored as secondary outcome. Another clinical outcome variable is the validated HEME bleeding score [40]. Also, photographs of the insertion site at different time points are blindly scored. Other clinical variables include ICU and hospital length of stay (LOS), ICU and hospital mortality, transfusion requirements and occurrence of transfusion reactions such as TRALI and allergic reactions. Next to descriptive statistics, the effect of prophylactic platelet transfusion on the primary and secondary outcomes will be analysed using multiple logistic regression for patient-, procedural-, catheter- and transfusion-related characteristics. In patients receiving platelet transfusion, patient- and transfusion-related factors will be analysed to assess the relation to post-transfusion platelet increment. To investigate the relationship between bleeding events and platelet count (post-transfusion platelet count if applicable) we divide platelet count into five groups: 10–20, 20–30, 30–40, 40–50 and above 50. *P* values of 0.05 will be accepted as statistically significant, values will be reported with and without correction for multiple testing.

Health-economic outcomes are calculated in the cost-effectiveness analysis, where costs per procedure-related bleeding event (primary clinical outcome) will be estimated. The economic evaluation will estimate costs (saved) per PC transfusion avoided. Results will be extrapolated to the national level to estimate the total impact on the health care budget per annum for the Netherlands in terms of cost reduction and increase in procedure-related bleeding events.

### Data collection

Inclusion is based on platelet count determined in the 24 h prior to randomisation. Baseline parameters, such as age, gender, height, weight and Body Mass Index (BMI), are collected. Recorded clinical parameters include date of admission, diagnosis, cause of thrombocytopenia, indication for CVC, use of defined confounding medication, hepatic- and kidney failure, diffuse intravascular coagulopathy and fever. For intensive care patients, also APACHE score, admission diagnosis and information about setting during admission are obtained. For haematologic patients, information about the haematologic condition and the phase of therapy is collected. In all patients, the most recent PT (INR), aPTT and haemoglobin levels, prior to randomisation are recorded. Information about the CVC, such as type, indication and diameter, is collected. Procedural details, such as insertion site, number of punctures, inadvertent arterial puncture, manual compression and the administration of ‘rescue platelets’, are recorded. According to standard care, blood is collected at 1 h and 24 h after CVC placement. In this way the effect of the platelet concentrate, if applicable, can be measured. A photographic image of the insertion site is taken prior to CVC placement, as well as directly post procedural and at 1 and 24 h after CVC placement. Clinical bleeding will be assessed at these same time points. All administered transfusion of blood products will be registered, as well as their clinical indication. All radiologic and surgical interventions will be documented.

### Follow-up

The follow-up time for the primary outcome is 24 h. Patient information about both ICU and hospital discharge or death, whichever comes first, are collected.

### Data management

An eCRF for the PACER trial in Open Clinica is developed. All participating centres have 24-h access to the eCRF. Data can be entered continuously for all participating patients. The principle investigator has access to the filled-out eCRFs. If data are entered incompletely or incorrectly, the principle investigator can contact the participating centres for clarification.

### Statistical considerations

We target to include 392 patients, with a potential limit of 462 patients to accommodate loss to follow-up and a possible dropout. The expected rate of severe peri-procedural bleeding incidents is around 0% [28, 33, 41–43]. Major bleeding is defined as WHO grade 3: gross blood loss requiring transfusion, or grade 4: debilitating blood loss (Additional file 1: Table S1). Considering the low complication occurrence, sample sizes that are by far unrealistic within the proposed study are required to demonstrate non-inferiority with an acceptable non-inferiority limit of (< 1%). We therefore also include WHO grade-2 bleedings in the definition of the primary outcome. As the observed bleeding events will concern predominantly grade-2 bleedings, and probably no or only incidental bleedings with more severe grades 3 or 4, an upper limit reflecting an absolute risk increase of 2.5% more bleeding events as the criterion to statistically demonstrate non-inferiority is considered both statistical and clinical acceptable. The sample size is increased by 2% to correct for loss to follow-up and by 15% to correct for dropouts, e.g. refusal of consent. Using a non-inferiority design, a sample size of 196 patients (per arm) and a percentage bleeding events of 1% in the control group and an expected percentage bleeding events of 1% in the experimental group will result in a power of 80% to exclude that there is a significant bleeding rate in the experimental arm (> 3.5% bleedings, absolute risk increase 2.5%, two group *t* test with a 0.05 two-sided significance level). As we do not anticipate any loss to follow-up – considering the short time-span of the study – we therefore intend to enrol 392 patients in total.

### Statistical analysis

Statistical analysis will be based both on an intention-to-treat principle and a per-protocol approach. Baseline assessments and outcome parameters will be summarised using simple descriptive statistics. The main analysis focusses on a comparison between the trial treatment groups of the primary outcome, the occurrence of relevant bleedings, expressed in a relative risk estimate and absolute risk increase, with the associated 95% upper confidence limit. Non-inferiority is demonstrated if this interval does not exceed the non-inferiority limit of 2.5% absolute difference in favour of transfusion.

### Withdrawal and replacement of individual patients

Subjects can leave the study at any time for any reason if they wish to do so without any consequences. The investigator can decide to withdraw a subject from the study for urgent medical reasons. When deferred consent is not obtained after randomisation and provisional inclusion of a patient, the randomised subject will be replaced. These cases will be recorded in the randomisation log without patient-specific data. The randomisation subject will be replaced in order to retain properly distributed randomisation groups and stratification.

### Study organisation

The Steering Committee is composed of the principal investigators, the coordinating investigator and the local investigators in the participating ICUs and haematology departments. The coordinating investigator is responsible for administrative management and communication with the local investigators and provides assistance to the participating clinical sites in trial management, record keeping and data management. The coordinating investigator helps in setting up local training in the participating centres to ensure the study is conducted according to the ICH-GCP guidelines, to guaranty integrity of data collection and to ensure timely completion of the case report forms. The local investigators provide structural and scientific leadership. They guarantee the integrity of data collection and ensure timely completion of the case report forms.

An independent monitor is installed to perform study monitoring. Remote monitoring by means of queries on the database will be done by a statistician and analysed by the monitor to signalise early aberrant patterns, trends, issues with consistency or credibility and other anomalies. On-site monitoring will comprise controlling presence and completeness of the research dossier and the informed consent forms, source data checks will be performed in the files of the first three patients of each participating centre, followed by at least 10% of files. Each centre will be visited at least once every year.

An independent Date Safety and Monitoring Board watches over the ethics of conducting the study in accordance with the declaration of Helsinki, monitors safety parameters and the overall conduct of the study. The DSMB is composed of three independent individuals (Dr. MGW Dijkgraaf, Dr. MCA Müller, Prof. Dr. JJ Zwaginga). The DSMB will meet by conference calls. The first took place before the first inclusion. Subsequent to this meeting the DSMB will meet approximately every 6 months. Also, a meeting will be scheduled after half of the total number of patients are enrolled.

As severe bleeding complications after central venous catheterisation are extremely rare, no bleeding-related serious adverse event is expected. All unexpected adverse events will be reported to the DSMB. Any report and/or advice of the DSMB will be send to the sponsor of the study, the Academic Medical Centre, Amsterdam, The Netherlands. Should the sponsor decide not to fully implement advices of the DSMB, the sponsor will send the advice to the reviewing Institutional Review Board, including a note to substantiate why (part of) the advice of the DSMB will not be followed.

## Discussion

This is the first randomised trial that investigates the safety of a lower platelet concentrate transfusion threshold prior to CVC placement, in a non-inferiority design. If this trial concludes that omission of prophylactic platelets in patients with a platelet count of 10 × 10^9^/L and higher is safe, guidelines that advise routine administration of platelet concentrate might be adjusted. For the Netherlands alone, we calculated that a transfusion threshold of 10 × 10^9^/L for prophylactic platelet transfusion prior CVC placement instead of 50 × 10^9^/L could lead to a cost reduction of 9.2 million euros per year [44].

Central venous catheterisation access is a frequently applied medical intervention; more than five million catheters are inserted in the United States each year [45, 46]. A peripherally inserted central catheter (PICC line) is often not an adequate alternative for percutaneous central vein cannulation because it bears an increased risk for thrombosis, especially in haemato-oncologic and critically ill patients [47].

Various factors associated with bleeding complications after CVC placement have been identified [29, 38, 48, 49]. The risk of bleeding after central venous catheterisation is multifactorial and is composed of procedural, patient and physician characteristics. Number of attempts, inadvertent arterial puncture, vein size, vein lesion, patient compliance, obesity and pulmonary hyperinflation have also been associated with bleeding risk [46, 50]. Therefore, it is difficult to assess the isolated effect of thrombocytopenia. Randomisation will divide patient-related factors evenly over both treatment arms. All lines are placed by experienced proceduralists, under real-time US guidance.

A concern regarding safety could be the occurrence of major bleeding in the no-platelet-concentrate-transfusion arm of the study. Based on previous observational studies we believe that the risk for serious complications is limited. We monitor the patients intensively and rescue platelet concentrate is available. Prolonged manual compression, or a suture at the insertion site are suggested as safe and minimally invasive interventions to stop prolonged bleeding [28, 42, 51]. In contrast, patients randomised to not receiving platelet concentrate are not exposed to the inherent risk of platelet transfusion.

A potential shortcoming of the current trial is the heterogeneous patient population having different causes of thrombocytopenia. Our study includes the two patient populations in which thrombocytopenia and central venous cannulisation are most frequent. We believe that this is also is a strength of our study, allowing results to be extrapolated to various patients categories.

In conclusion, this is the first prospective, randomised controlled, non-inferiority trial powered to test the hypothesis whether not correcting thrombocytopenia prior to central venous cannulation does not lead to an increased occurrence of bleeding complications in critically ill and haematologic patients.

## Trial status

Currently recruiting

## Additional files

Additional file 1: **Table S1.** WHO bleeding score. Specified for central venous catheter-related bleeding. All bleeding must be central venous catheter related, within 24 h after insertion. (DOC 29 kb)

Additional file 2: SPIRIT 2013 Checklist: recommended items to address in a clinical trial protocol and related documents. (PDF 119 kb)
