# Supplementary material for: Prophylactic platelet transfusion prior to central venous catheter placement in patients with thrombocytopenia: study protocol for a randomised controlled trial
Source: Trials. 2018 Feb 20;19:127. doi: 10.1186/s13063-018-2480-3 (PMC5819660; doi:10.1186/s13063-018-2480-3)
Supplement: Supplementary file 1 — Table S1. WHO bleeding score. Specified for central venous catheter-related bleeding. All bleeding must be central venous catheter related, within 24 h after insertion. (DOC 29 kb) [file 13063_2018_2480_MOESM1_ESM.doc]

Additional file 1: Table S1

| **WHO Bleeding score**  Specified for Central Venous Catheter related bleeding* | |
| --- | --- |
| Grade 0 | No bleeding. |
| Grade 1 | Oozing. Hematoma. Bleeding that requires less than 20 minutes of manual compression to stop. |
| Grade 2 | Bleeding that requires minor interventions to stop, such as prolonged manual compression (>20 minutes). |
| Grade 3 | Bleeding requiring radiologic, or elective operative interventions, red cell transfusion without hemodynamic instability. |
| Grade 4 | Bleeding associated with severe hemodynamic instability (hypotension; >50mm/Hg fall or >50% decrease in either systolic or diastolic blood pressure, with associated tachycardia (heart rate increase of > 20% for 20 minutes) and requiring RBC transfusion over routine transfusion needs or fatal bleeding. |

**All bleeding must be Central Venous Catheter related, within 24 hours after insertion.*
